# Perinatal depression and associated factors among reproductive aged group women at Goba and Robe Town of Bale Zone, Oromia Region, South East Ethiopia

**DOI:** 10.1186/s40748-015-0013-6

**Published:** 2015-05-14

**Authors:** Tomas Benti Tefera, Asfaw Negero Erena, Kemal Ahmed Kuti, Mohammedawel Abduku Hussen

**Affiliations:** Department of Nursing, Madawalabu University, College of Medicine and Health Sciences, Bale-Goba, South east Ethiopia; Department of Medicine, Madawalabu University, College of Medicine and Health Sciences, Bale-Goba, South east Ethiopia; Department of Public Health, Madawalabu University, College Of Medicine and Health Sciences, Bale-Goba, South east Ethiopia; Department of Midwifery, Madawalabu University, College Of Medicine and Health Sciences, Bale-Goba, South east Ethiopia

**Keywords:** Maternal depression, Mental Health, Pregnancy, Perinatal depression

## Abstract

**Background:**

In sub Saharan Africa little progress has been made towards achieving the Millennium Development Goals. Lack of achievement of MDGs is reflected in only minor changes in maternal mortality and child health – this is especially true in Ethiopia. Perinatal depression is common in developing countries where one in three women has a significant mental health problem during pregnancy and after childbirth. Perinatal depression is associated with inadequate prenatal care and poor maternal weight gain, low infant birth weight, and infant growth restriction. This study determined the prevalence of perinatal depression and its associated factors among reproductive age group women at Goba and Robe town of Bale zone; Oromia Region, South East Ethiopia. A cross sectional study with Simple Random sampling was employed to include 340 eligible subjects. The WHO self reporting questionnaire with 20 items with a cut off point 6 and above was used to separate non-cases/cases of perinatal depression. Data were collected by trained data collectors. Descriptive analysis was done using SPSS Version 16. Multivariate logistic regression was used to identify independent predictors of perinatal depression at 95% CI and P value of ≤ 0.05.

**Results:**

Prevalence of perinatal depression was about 107(31.5%). About 20(5.9%), 86(25.3%) were current smokers and alcohol consumers respectively. Two hundred seventy seven (71.2%) of the respondents reported husband support during their pregnancy and after birth and 195(59.3%) were reported support from the husband’s family/relatives. Maternal perceived difficulty of child care, family History of mental illness, family visit during the perinatal period, history of child death and husband smoking status were found as independent predictors of perinatal depression.

**Conclusion:**

This study found that 1 in 3 women in this region of Ethiopia have depression. Depression screening is not currently routine care, but should be given due attention due to the high prevalence of depression in these populations. Public health agencies could organize special training events for Health care workers, including Health Extension workers on Mental Health and has to provide screening service to strengthen mental health in the pregnant and postpartum family.

## Background

The perinatal period typically refers to the time from conception to the end of the first postpartum year [1]. This is a time when the woman’s life is associated with profound physical and emotional changes, and associated risks for the onset or exacerbation of several mental disorders [2]. One of the most common mental health problems occurring in women during their childbearing years is depression. Perinatal depression refers to major and minor depressive episodes that occur either during pregnancy or after delivery [1].

Perinatal depression is common in developing countries [3] and one in three women has a significant mental health problem [4]. This problem is a serious but under-recognized public health problem in low and middle income countries making a substantial contribution to maternal and infant morbidity and mortality. About 12.5 - 42% of pregnant women and, 12 - 50% of mothers of newborns in low and middle income countries such as Ethiopia screen were positive for symptoms of depression [5].

There is evidence indicating that maternal common mental disorders, in particular depressive disorders poses a serious public health concern because of their adverse effect on infant development [6] like poor nutrition, stunting, early cessation of breastfeeding, diarrhoeal disease [7].

Pregnant women or mothers with mental health problems often have poor physical health and also have persistent high-risk behaviours including alcohol and substance abuse. They have increased risk of obstetric complications and preterm labour because they are less likely to seek and receive antenatal or postnatal care or adhere to prescribed health regimens [8].

It was also reported that in sub Saharan Africa little progress has been made towards achieving the Millennium Development Goals, In particular little improvement is reflected with rates of maternal mortality and child health which is also true in Ethiopia [9].

A community based study conducted among mothers revealed that the overall prevalence of maternal depression was about 33% in Ethiopia [10]; however, this study did not included pregnant women rather women having a Childs of age 6-18months.

So studying the mental depression during perinatal period and its associated factors are very important to provide crucial information’s for different stake holders improve maternal mental health services and tackle its grave consequences on child growth and development who are the future of our country and also incorporation of mental health services in maternal health services which in turn reduce maternal mortality rate and its severe consequences. This study was aimed to determine prevalence’s of perinatal depression and associated factors among reproductive age group women at Goba and Robe Town of Bale zone, Oromia Region, South East Ethiopia.

## Methods

### Study area and period

Bale is one of the zones in the Oromia region of Ethiopia. The zone has three administrative towns which are Goba town, Robe town and Ginner town. The study area has two hospitals; health centers and health posts. The study period was from March to April 2014.

### Study design

Community based Cross-sectional survey was employed.

### Source population

All pregnant women and women with child under one year of age (at postpartum period) in the selected kebele of Goba and Robe town.

### Study population

Pregnant women and women with Under 1 yr of child from the source population.

### Sample size determinations

Sample size was determined based on a single proportion formula considering 33% prevalence of maternal mental disorder in Ethiopia [10], 95% confidence level and 5% marginal error. The final sample size by including 5% non-response rate was become 357.

### Sampling procedures

First three kebeles [East Goba from Goba town, Baha Biftu from Robe town and west Goba] were selected from both towns randomly. Then households with pregnant women and having less than one year in these kebele were identified and sampling frame was prepared. Finally Simple Random sampling was used and eligible subjects interviewed. In cases of more than one eligible subject in selected household one of them was selected by lottery methods.

### Study variables

Independent variables included in this study were Maternal Socio economic-demographic variables, Maternal and family history of mental illness, Maternal current alcohol consumption and Tobacco uses, Support during pregnancy and child birth, Families visit during pregnancy and child birth, Support during perinatal period from families/relatives, Age at marriage, plan to have child, Number of total & female children, Husband educational level, employment, husband uses of tobacco and alcohol, Husband history of chronic medical diseases/Hypertension, diabetes Mellitus and any others) and Previously experienced death of one of own children and the Dependent Variable was a perinatal depression.

### Data collection tool and data collection procedures

The WHO, Self Reported Questionnaire with 20 items was used to assess the perinatal depression. The tool consists of twenty Yes/No questions with a reference period of the previous thirty days. It has acceptable levels of reliability and validity in developing countries and is recommended by the World Health Organization as a screening tool for depression [11].

A semi-structured, pre-tested and interviewer administered questionnaire in Amharic language was used to collect data. The tool also includes maternal and husband socio demographic variables, maternal history of pervious mental illness, family history of mental diseases, and the presence of social support for during the perinatal period, Husband and maternal Tobacco use and alcohol uses in last 12 months, and Obstetric history like Age at marriage, Numbers of total children, plan to have child, experiences of the deaths of one of own child.

A study conducted in ethiopia validated SRQ-20 for use in a mixed sample of pregnant and postnatal women in the Butajira population and concluded that the overall performance of the SRQ did not differ significantly between pregnant and postnatal women and the study concluded 6 and above as cut off point to differentiate the cases from non cases [12].

So for this study a cutoff point six and over was used to differentiate cases and non cases of perinatal depression.

Data were collected through face to face interviews by 10 urban Health extension workers for the duration of 15 days after getting Intensive training on sampling procedures and how to approach and conduct the interview with the study subjects.

### Data analysis

Data analysis was conducted using the Social Sciences (SPSS) Version 19.0 for windows. Descriptive analysis was first done on the total sample. Perinatal women were then classified as having depression based on a score greater than or equal to six on the SRQ. Bivariate logistic regression was used to identify associated independent variables to cases of depression. Then, Variables significantly associated on Binary Logistic regression (P ≤ 0.05) were included in Multiple Logistic regression to identify independent predictors of perinatal depression. Statistical significance was declared at P ≤ 0.05.

#### Data quality assurance

Pretest was performed on the subjects other than study subjects to check some ambiguous questions and unclear question and necessary amendment was made. Intensive training was given to data collectors regarding the interview technique and subject selection for interview. The collected data were reviewed and checked for completeness on the day of each data collection by assigning supervisors.

### Ethical consideration

Ethical clearance was obtained from the Madawalabu University Research and community service directorate. The zonal health office was asked for permission to conduct the study. Then after the permission letter was given to respective town health offices. After permission obtained from town health offices the actual study was commenced. Informed consent was obtained from each study subjects after clarifying the about the purpose of the study & other relevant information about the study. Privacy and confidentiality were strictly maintained.

## Results

From the total 357 perinatal women, 340 of them were included in the study yielding the response rate of 95.2%. The mean age of the study subjects was 22.9(SD ± 2.1). Of the total respondents 258(75.9%) were literate in educational status, 190(55.9%) were Christian in religion, 180(52.9%) were at 25–34 age group (Table 1).

**Table 1:** **Distribution of study subjects by Socio demographic characteristics, 2014**

**Socio demography variables**	**Responses**	**Number**	**Percentage**
Educational status	Illiterate	82	24.1
	Literate	258	75.9
Religion	Muslim	150	44.1
	Christian	190	55.9
Occupation	House wife	205	60.3
	Farmer	26	7.6
	Merchant	35	10.3
	Employee	59	17.4
	Other**	15	4.4
Marital status	Single	21	6.2
	Married	251	73.8
	divorced	57	16.8
	Widowed	9	2.6
Age	15-24	92	27.1
	25-34	180	52.9
	35 and above	68	20.0
Perceived difficult of child care	Yes	108	31.8
	No	232	68.2
Confident r/ship with husband family	yes	220	64.7
	No	120	35.3

** includes students, daily labourer, and tella makers.

Three hundred twenty two (94.7%) of Respondents reported previous history of mental illness and 323(95%) were had family history of mental illness. Nearly six percent of respondents were current smokers and 86(25.3%) were current alcohol consumers (Table 2).

**Table 2:** **Distribution of study subjects on history of mental illness and maternal substance uses, 2014**

		**Number**	**Percentage**
History of mental illness	Yes	18	5.30
	No	322	94.7
Family history of mental illness	Yes	17	5.0
	No	323	95.0
History of medical diseases	Yes	35	10.3
	No	305	89.7
Smoking tobacco	Yes	20	5.9
	No	320	94.1
Alcohol consumption	Yes	86	25.3
	No	254	74.4

### Husband or Marital factors

About 278(87.1%) were reported that their husband were literate in educational status. Two hundred seventy seven (71.2%) of the respondents reported that their husband provide any support during their pregnancy and after birth too while the rest not. Husband psychological abuse and physical abuse were also asked and about 57(17.9%), 79(24.8%) were reported the abuse respectively. About 137(42.9%) of study subjects reported their husband used substances. About 195(59.3%) were reported support from family/relatives and about 213(64.3%) reported visit from their families/ relatives during pregnancy and after births at least two and more times (Table 3).

**Table 3:** **Frequency distributions of husband marital factors, 2014**

		**Number**	**Percentage**
Educational status of husband	Literate	278	87.1
	Illiterate	41	12.9
Support during pregnancy and after birth	Yes	227	71.2
	No	92	28.8
Physical abuse	Yes	57	17.9
	No	262	82.1
Psychological abuse	Yes	79	24.8
	No	240	75.2
Husband alcohol use	Yes	137	42.9
	No	182	57.1
Husband smoking	Yes	63	19.7
	No	256	80.3
Support from family or relatives	Yes	195	59.3
	No	134	40.7
Family/relative visit during perinatal period	Yes	213	64.5
	No	117	35.5

### Obstetric factors

The mean age at marriage was 21.5 ±SD 3.5. Of the total respondents involved in this study, about 45(13.3%) were had no children, 150(44.2%) were had two to four children’s and 38(11.2%) were had above four children. Concerning their pregnancies status about 120(35.6%) were reported it was not planned and the rest otherwise. About 34(12%) were reported they had experienced death of own child (Table 4).

**Table 4:** **Distribution of study subjects on selected obstetric factors, 2014**

	**Number**	**Percentage**
Total number of children		
No children	45	13.3
Only one	106	31.3
Two- to four	150	44.2
Above five	38	11.2
Total number of female children		
No female children	60	20.4
Only one female children	137	46.6
Above two female children	97	33.0
Experience of own child death		
Yes	34	12.0
No	250	88.0
Was planned to be pregnant or to have this child		
Yes	217	64.4
No	120	35.6
Mean Age at marriage	Mean 21.5 ± SD3.5	

### Prevalence of perinatal depression

One Hundred Seven (31.5%) were classified as having perinatal depression and about 233(68.5%) were classified as not having perinatal depression.

### Factors associated with perinatal depression among the study subjects

The women who had no perceived difficulty of child care and No husband tobacco users were less likely to have perinatal depression compared to their counterparts, (COR = 0.32, 95%CI (0.19-0.51), (COR = 0.32, 95%CI (0.18-0.57) respectively. The odds of having depression among women who had no family/relative support, no family visit and un-planned pregnancy were more likely compared to their counterparts (Table 5).

**Table 5:** **Binary logistic regressions: predictors of perinatal depression among study subjects, 2014**

**Variables**	**No perinatal depression**	**Perinatal depression**	**COR**	**P**	**95%CI**
Educational status of women					
Illiterate	40(48.8%)	42(51.2%)	3.11	0.000	1.86-5.22
Literate	193(74.8%)	65(25.2%)	1		
History of mental illness					
No	226(70.2%)	96(29.8%)	0.27	0.009	0.10-0.72
yes	7(38.9%)	11(61.1%)	1		
History of family mental illness					
No	226(70.0%)	97(30.0%)	0.30	0.018	0.11-0.81
yes	7(41.2%)	10(58.8%)	1		
Perceived difficulty of child care					
No	178(76.7%)	54(23.3%)	0.32	0.000	0.19-0.51
yes	55(50.9%)	53(49.1%)	1		
Confident with your husband or other relatives					
No	62(51.7%)	58(48.3%)	3.27	0.000	2.02-5.27
Yes	171(77.7%)	49(22.3%)	1		
Living with your husband					
No	17(36.2%)	30(63.8%)	4.95	0.000	2.58-9.48
Yes	216(68.5%)	77(26.3%)	1		
Husband educational status					
Illiterate	34(54.8%)	28(45.2%)	2.16	0.022	1.12-4.17
Primary	22(53.7%)	19(46.3%)	2.26	0.032	1.07-4.78
Secondary	101(76.5%)	31(23.5%)	0.80	0.468	0.45-1.45
TVET& above	76(72.4%)	29(27.6%)	1		
Husband tobacco uses					
No	195(76.2%)	61(23.8%)	0.32	0.000	0.18-0.57
Yes	32(50.8%)	31(49.2%)	1		
Support from relatives					
No	68(50.7%)	66(49.3%)	4.76	0.000	2.87-7.89
Yes	162(83.1%)	33(16.9%)	1		
Family visits					
No	55(47.0%)	62(53.0%)	5.36	0.000	3.22-8.91
Yes	176(82.6%)	37(17.4%)	1		
Pregnancy status					
Not planned	68(56.7%)	52(43.3%)	2.36	0.000	1.47-3.81
Planned	164(75.6%)	53(24.4%)	1		
Experience of child death					
No	188(75.2%)	62(24.8%)	0.18	0.000	0.08-0.39
Yes	12(35.3%)	22(64.7%)	1		
Total number of child					
No children	27(60.0%)	18(40.0%)	0.74	0.50	0.31-1.77
one child	87(82.1%)	19(17.9%)	0.24	0.001	0.11-0.54
2- 4 child’s	98(65.3%)	52(34.7%)	0.59	0.150	0.29-1.21
≥5 child’s	21(53.8%)	18(46.2%)	1		

TVET: technical and vocational education training. AOR: adjusted Odd Ratio, P: P Value.

Result from multivariate analysis revealed that Maternal perceived difficulty of child care, Family History of mental illness, family visit during the perinatal period, history of child death and husband smoking status were found as independent predictors of perinatal depression. The odds of having depression among women/mothers who hadn’t experienced adverse life event (death of own child) were less likely compared to their counterparts (AOR = 0.30, 95%CI (0.11-0.86) (Table 6).

**Table 6:** **Multiple logistic regressions: predictors of perinatal depression among study subjects, 2014**

**Variables**	**No perinatal depression**	**Perinatal depression**	**AOR**	**P**	**95%CI**
Educational status of women					
Illiterate	40(48.8%)	42(51.2%)	2.25	.053	0.98-5.15
Literate	193(74.8%)	65(25.2%)	1		
History of mental illness					
No	226(70.2%)	96(29.8%)	0.55	.445	0.12-2.53
yes	7(38.9%)	11(61.1%)	1		
History of family mental illness					
No	226(70.0%)	97(30.0%)	0.17	.042	0.03-0.92
yes	7(41.2%)	10(58.8%)	1		
Perceived difficulty of child care					
No	178(76.7%)	54(23.3%)	0.43	.031	0.20-0.93
yes	55(50.9%)	53(49.1%)	1		
Confident with your husband or other relatives					
No	62(51.7%)	58(48.3%)	1.03	.058	0.38-0.15
Yes	171(77.7%)	49(22.3%)	1		
Living with your husband					
No	17(36.2%)	30(63.8%)	2.08	.255	0.58-7.37
Yes	216(68.5%)	77(26.3%)	1		
Husband educational status					
Illiterate	34(54.8%)	28(45.2%)	0.54	.290	0.17-1.69
primary	22(53.7%)	19(46.3%)	0.48	.226	0.15-1.57
secondary	101(76.5%)	31(23.5%)	0.46	.095	0.16-1.15
TVET& above	76(72.4%)	29(27.6%)	1		
Husband tobacco uses					
No	195(76.2%)	61(23.8%)	0.28	.004	0.12-0.67
Yes	32(50.8%)	31(49.2%)	1		
Support from relatives					
No	68(50.7%)	66(49.3%)	3.25	.031	1.11-9.52
Yes	162(83.1%)	33(16.9%)	1		
Family visits					
No	55(47.0%)	62(53.0%)	1.52	.500	0.45-5.06
Yes	176(82.6%)	37(17.4%)	1		
Pregnancy status					
Not planned	68(56.7%)	52(43.3%)	1.02	.960	0.46-2.24
Planned	164(75.6%)	53(24.4%)	1		
Experience of Child death					
No	188(75.2%)	62(24.8%)	0.30	.025	0.11-0.86
Yes	12(35.3%)	22(64.7%)	1		
Total number of child					
No children	27(60.0%)	18(40.0%)	0.71	.743	0.09-5.41
one child	87(82.1%)	19(17.9%)	0.52	.316	0.14-1.87
2- 4 child’s	98(65.3%)	52(34.7%)	1.44	.490	0.51-4.09
≥5 child’s	21(53.8%)	18(46.2%)	1		

TVET: technical and vocational education training. AOR: adjusted Odd Ratio, P: P Value.

The odds of having depression among women/mothers who hadn’t supported from families were more likely compared to their counterparts (AOR = 3.25, 95%CI (1.11-9.52).

## Discussion

Although pregnancy and childbirth are generally viewed as a joyful time to most families, they also put women at risk of developing mental problems-perinatal depression due to different factors. The prevalence of perinatal depression varies across the region in the world. These differences might be due to differences in the type of instrument and cutoff score used, cultural variables, differences in perception of mental health, differences in socioeconomic environments, levels of social support or its perception, as well as biological vulnerability factors [1].

The perinatal period is also a high risk time for the emergence of depressive symptoms [13] and these depressive symptoms can be efficiently measured using screening questionnaires which can be used as proxy measures of depressive disorder after validation against Gold standard tools.

For this particular study, WHO Self Reporting Questionnaire, a 20-item questionnaire which is validated for use developing countries such as, Ethiopia.

Recent studies on low and middle income countries reported that on the prevalence of maternal mental problem ranges from 10-41%, depending on the place and time of the perinatal period studied and the instruments employed [5]. This study also found that the prevalence of perinatal depression was about 31.5%) which is in a specified range of the problem in low and middle income countries.

A community based study conducted among mothers using SRQ instrument revealed that the overall prevalence of maternal depression was about 30% in India and 30% in Peru with cut off point seven and above [10]. This study also found comparable finding but higher than the prevalence of depression in Indonesia which was about 22% [14]. However the later study was conducted using EPDS with cut off point of 12 and above as having postnatal depression.

A research conducted by Rochat et al. among pregnant women of South Africa using the EPDS found that the prevalence of depression was about 41% which was higher than the finding of this study. The possible reason may be the difference in the lifestyle of the study participants and the instrument used [15].

Another study conducted in Ethiopia revealed that the prevalence of perinatal depression was about 33%. This finding is comparable with the finding from this study. It may be due to that the lifestyle, culture and the health systems of the country similar [10].

Depression has some identifiable risk factors. Depression may result from poor environmental factors, such as lack of food, inadequate housing, little financial support, and insufficient family support (e.g., an uninvolved husband or partner) [16].

Social support was demonstrated to be important in the transition to motherhood and has an impact on emotional coping [17]. It gives direct effects on emotional stability, attenuated effects of stressful life events, and prevents depression [18]. A Study conducted among Thai women revealed that Lack of social support as an independent predictor for postpartum depression [19]. Another study conducted in Indonesia also showed that lack of support from husband was associated with maternal perinatal depression during pregnancy [20]. The current study also revealed that not having support from families or relatives during perinatal period was found as an independent predictor of perinatal depression. This is due to that, not having the social support makes them vulnerable to stress, worthlessness, and hopelessness [21].

Different studies found that history of pregnancy loss, stressful life events (death of a child) and financial difficulties were associated with maternal depression [22,23]. The current study also found that perceived difficulty of child care and experience of child death was significant predictors of perinatal depression.

Most women receive some form of prenatal care, making several visits to see perinatal professionals during the course of pregnancy and after childbirth. These visits present a unique opportunity for health care professionals for an early identification of maternal depression. This finding also helps policy makers to build a comprehensive network of community perinatal services and service providers to strengthen mental health of the pregnant and postpartum family.

This social network might provide a range of supports such as parent education and child development issues, postpartum home visits, nutritional counselling, education on Mental Health problems and its sign and symptoms to seek health care for mothers and other family members. These services can be provided by Health Extension workers so that-the risks of maternal health problems and its consequences intervened.

The current study tried to determine the perinatal depression and its associated factors that are amenable to change. As the strength, Study used Validated WHO SRQ-20 Questionnaire in Ethiopian context and Data were collected by trained urban Health extension workers. The limitations were- the study not addressed biological factors- hormonal and chemical changes, experienced by women during the perinatal period and the HIV/AIDS status of the women. Since it is Cross sectional study design, cause-effect relationship cannot be determined and longitudinal prospective research is needed to fully understand the nature of these factors in perinatal depression.

## Conclusion

This study found that 1 in 3 women in this region of Ethiopia have depression. Depression screening is not currently routine care, but should be given due attention due to the high prevalence of depression in these populations.

Public health agencies could organize special training events for practitioners and support staff within the maternal and child health profession. Health Extension workers have to be trained on Mental Health problem detection/screen/ and has to provide service to strengthen mental health of the pregnant and postpartum family.

## Abbreviations

CES-D: Center of Epidemiological Studies-Depression scale
CI: Confidence interval
CMD: Common mental disorder
DALYs: Disability adjusted life years
EDHS: Ethiopia Demographic Health Survey
EPDS: Edinburgh postnatal depression syndrome
HADS: Hospital anxiety and depression scale
LMICs: Low and middle income countries
MDG: Millennium developmental goal
PTSD: Post traumatic syndrome disorder
SPSS: Statistical package for social sciences
SSA: Sub Saharan Africa
SRQ: Self reported questionnaire
WHO: World Health Organization
